# Postoperative Pain Management in Patients with Lower-Extremity Amputations: A Single-Institution Retrospective Analysis of the Effectiveness of Non-opioid Analgesics in Reduction of Opioid Use

**DOI:** 10.7759/cureus.98536

**Published:** 2025-12-05

**Authors:** Chandler H Dinh, Hyun Ah Park, Andrew Y Vassantachart, Navdeep S Manhas, Duc A Tran, Eugene Pak

**Affiliations:** 1 Physical Medicine &amp; Rehabilitation, Loma Linda University School of Medicine, Loma Linda, USA; 2 Physical Medicine &amp; Rehabilitation, Loma Linda University Medical Center, Loma Linda, USA; 3 Pain Medicine, Loma Linda University Medical Center, Loma Linda, USA

**Keywords:** amputation pain, gabapentin, inpatient rehabilitation facility, lower extremity amputation, morphine milligram equivalents, multimodal analgesia, non-opioid analgesics, opioid reduction, postoperative pain management, pregabalin

## Abstract

Background: Pain management following lower extremity amputation remains a clinical challenge, particularly during inpatient rehabilitation. Although opioids are frequently used, their adverse effects and potential for prolonged use highlight the importance of multimodal pain strategies. This study investigated the relationship between specific non-opioid analgesics and reductions in opioid use among patients undergoing inpatient rehabilitation after major lower extremity amputation.

Methods: A retrospective review was conducted at a single inpatient rehabilitation facility (IRF), examining opioid utilization measured in morphine milligram equivalents (MME) and the concurrent use of non-opioid agents, including acetaminophen, methocarbamol, gabapentin, pregabalin, and duloxetine. Eighty-one patients admitted between January 2021 and December 2023 were included. Admission and discharge opioid requirements were compared with daily non-opioid doses. Statistical analyses were performed using STATA version 3 (StataCorp LLC, College Station, TX).

Results: Gabapentin use was associated with a significant decrease in MME from admission to discharge (p=0.04), with an average daily dose of 1000 mg corresponding to a reduction of 1 MME per day. In multivariable analysis incorporating all non-opioid medications, gabapentin and pregabalin both showed significant associations with reduced MME (p=0.021 and p=0.027, respectively).

Conclusion: Among patients recovering from lower extremity amputation during inpatient rehabilitation, gabapentin use was significantly associated with lower opioid requirements, with pregabalin demonstrating a similar pattern. Incorporating these agents into multimodal analgesic regimens may help optimize postoperative pain control and limit opioid exposure. Further research is warranted to explore the role of additional non-opioid options in this setting.

## Introduction

In the United States, more than 185,000 individuals undergo upper or lower amputations each year, and the number of people with an amputation is projected to double by 2050 due to increasing rates of vascular disease and related sequelae [1]. Among the issues that affect patients after amputation in both the acute and chronic postoperative periods is post-amputation pain, which includes phantom pain and residual limb pain [2].

Many lower extremity amputation patients are admitted to an inpatient rehabilitation facility (IRF), where opioids are often used in the pain regimen. Clinicians must balance postoperative pain management with the side effects and risks of analgesic use. For example, phantom limb pain, which is estimated to occur in up to 83% of patients who have undergone amputation, can impair quality of life, hinder rehabilitation, and reduce return-to-work outcomes [3]. Opioid side effects include nausea, vomiting, drowsiness, and delirium, which may contribute to increased mortality, reduced functional ability, and longer hospital stays [4]. Given the role of opioid medications in IRF pain regimens, it is important to consider the role IRFs may play in the development of chronic opioid usage. A 2021 single-center study investigating opioid use during an IRF admission found that for patients who utilized opioids, there was no statistical decrease in utilization between admission and discharge [5]. Another study analyzed 2,247 opioid-naïve, first-time lower extremity amputees in a national database and found that 44.6% of these patients had prolonged postoperative opioid use, defined as at least one prescription between 90 and 180 days [2]. Several studies have suggested that lower extremity amputees are at higher risk for developing chronic pain, as well as suffering opioid overdose [6,7].

With the rise of the opioid epidemic, there has been greater interest in multimodal analgesic strategies for postoperative pain management [8]. Multimodal pain management includes non-opioid medications such as anticonvulsants (e.g., gabapentin and pregabalin), non-steroidal anti-inflammatory drugs, and acetaminophen. Studies have demonstrated that utilizing non-opioid analgesics can decrease opioid use in patient populations such as critically ill trauma patients or those with traumatic brain injury in an IRF setting [9,10].

As highlighted above, patients who have undergone lower limb amputations represent an increasing patient population, with many undergoing rehabilitation at IRFs. Given the side effects of acute and chronic opioid usage, it is important to identify non-opioid analgesics in the amputee population that can reduce IRF opioid use. The aim of this retrospective review was to evaluate specific non-opioid analgesics that have the potential to reduce admission-to-discharge opioid usage in patients who have undergone lower limb amputations during their IRF hospitalization.

## Materials and methods

Study design

This retrospective review was approved by the Loma Linda University Institutional Review Board (IRB) with Human Research and Compliance and given the designation IRB# 5240398. Patients were selected from a database of Loma Linda University Acute Inpatient Rehabilitation, Loma Linda, CA, USA, and were included if they met the following criteria: (1) admission between January 1, 2021, and December 31, 2023; (2) a major lower extremity amputation (transfemoral or transtibial) within the last month; and (3) successful discharge from the inpatient service to home or a lower-acuity facility. Patients were excluded if they were prematurely transferred to an acute hospital for any reason. Patient confidentiality was maintained by creating a de-identified database that was accessed from protected computers on the hospital network.

Variables

The following data were obtained and recorded for each patient from the medical record: gender, age, length of stay (defined as the total number of full 24-hour days), type of amputation, daily morphine milligram equivalents (MMEs) on admission, and daily MMEs on discharge. For non-opioid analgesics, the following information was collected: daily dose on admission, daily dose on discharge, and average daily dose. Non-opioid medications included acetaminophen, duloxetine, gabapentin, pregabalin, non-steroidal anti-inflammatory drugs, lidocaine patch, amitriptyline, nortriptyline, baclofen, methocarbamol, cyclobenzaprine, tizanidine, venlafaxine, and diclofenac gel.

Statistical analysis

Power analysis was performed using G*Power 3.1 (Heinrich Heine University, Düsseldorf, Germany) with a 5% type I error based on data from King et al. [10]. The estimated sample size was 40 participants. All data were analyzed using STATA version 3 (StataCorp LLC, College Station, TX). Individual and multiple linear regressions comparing the average doses of non-opioid medications to the normalized change in admission-to-discharge MMEs were performed. A p-value < 0.05 was considered statistically significant.

## Results

**Figure 1:**
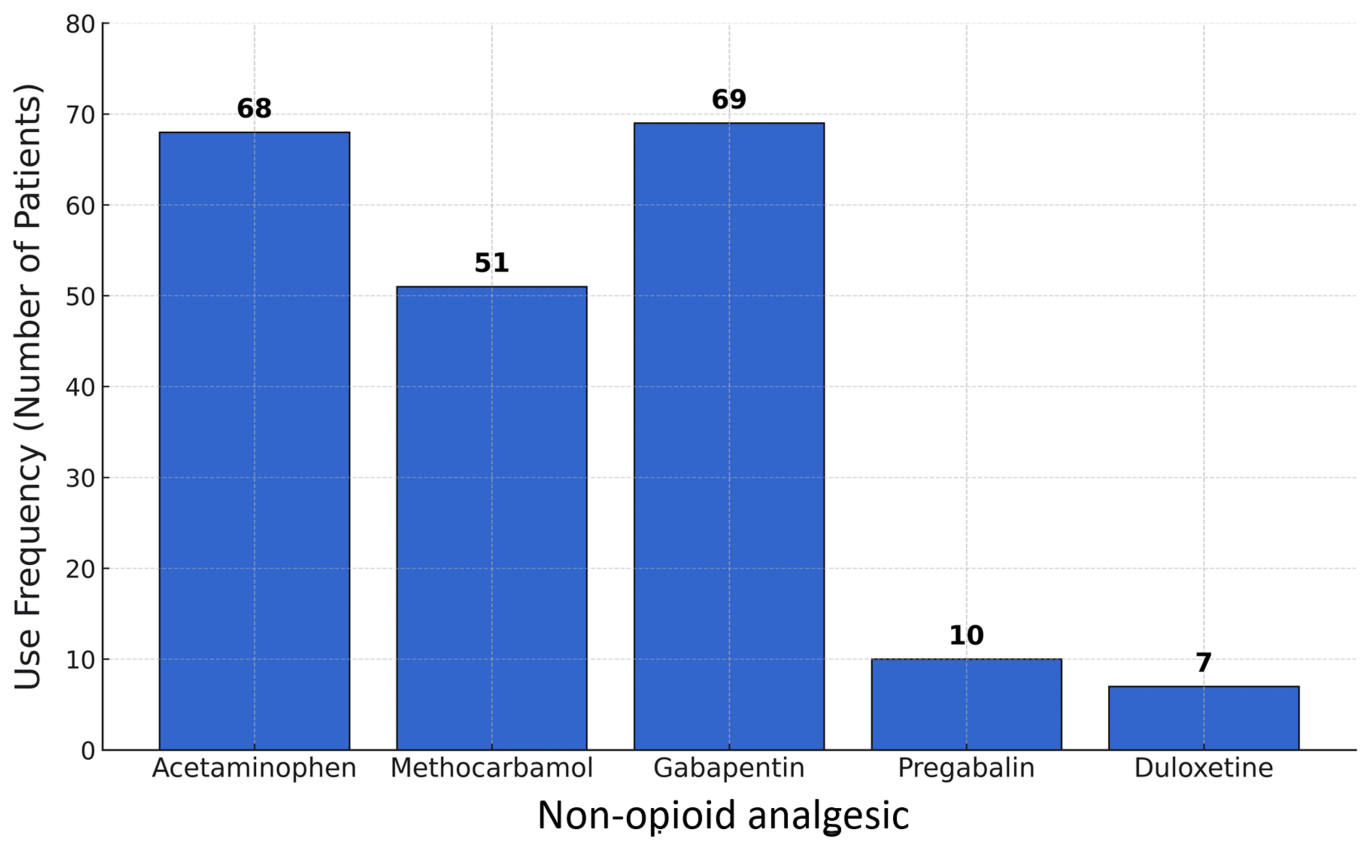
Non-opioid analgesic utilization

A total of 112 patients who had undergone lower-extremity amputation were admitted to the rehabilitation hospital during the study period, with 81 patients meeting the inclusion and exclusion criteria. The most common reason for exclusion was medical decompensation requiring higher-level medical care. Of all non-opioid analgesics prescribed, acetaminophen, methocarbamol, gabapentin, pregabalin, and duloxetine were the most frequently used (Figure 1).

Patient characteristics

Among the included patients, ages ranged from 22 to 87 years (mean, 55.7 years). Of the 81 patients, 54 were male, and 27 were female. Fifteen patients had a transfemoral amputation, and 66 had a transtibial amputation. Length of stay ranged from two to 49 days (mean, 13.8 days) (Table 1).

**Table 1:** Patient characteristics

Variable	Value
Age (years)	
Mean	55.9
Range	22 - 87
Sex (%)	
Male	66.7
Female	33.3
Amputation level	
Transfemoral	15
Transtibial	66
Length of stay (days)	
Mean	14
Range	2 - 49

Non-opioid analgesic usage

Multiple linear regression analysis comparing acetaminophen, methocarbamol, pregabalin, and duloxetine with normalized admission-to-discharge MME change demonstrated a significant reduction in MME with gabapentin (p = 0.021, r(75) = 0.001) and pregabalin (p = 0.027, r(75) = 0.015).

Individual regression analysis of the non-opioid analgesics revealed that the average daily dose of gabapentin (mg) significantly affected normalized MME (p = 0.041, r(79) = 0.001). No significant effect was observed for the average daily doses of acetaminophen, methocarbamol, pregabalin, or duloxetine (Table 2).

**Table 2:** Multiple and individual linear regression analyses comparing average daily dose of non-opioid analgesics to the normalized admission-to-discharge MME change MME: morphine milligram equivalents

Type of analysis	Non-opioid analgesic	Coefficient	P-value	95% CI
Multiple linear regression	Acetaminophen	-0.001	p=0.634	-0.0007-0.0004
	Duloxetine	0.0038	p=0.944	-0.1036-0.1112
	Gabapentin	0.0012	p=0.019	0.0002-0.0023
	Methocarbamol	-0.002	p=0.515	-0.0010-0.0005
	Pregabalin	0.0167	p=0.027	0.0024-0.0309
Individual regression	Acetaminophen	-0.0001	p=0.738	-0.0007-0.0005
	Duloxetine	0.618	p=0.618	-0.0820-0.1370
	Gabapentin	0.0010	p=0.041	0.00004-0.0021
	Methocarbamol	0.0001	p=0.795	-0.0007-0.0009
	Pregabalin	0.135	p=0.055	-0.0003-0.0274

## Discussion

Amputations are an increasingly common medical issue in the United States, and identifying effective postoperative pain management strategies is important [1]. While opioid medications are effective analgesics, they have undesired side effects, including gastrointestinal, cognitive, and addiction-related complications, which can limit their short- and long-term utility [2-4]. The goal of this study was to identify non-opioid analgesics, specifically acetaminophen, gabapentin, methocarbamol, pregabalin, and duloxetine, that could potentially reduce opioid burden in lower extremity amputees during their IRF hospitalization.

This retrospective study found that gabapentin had a significant effect on the average change in MMEs from admission to discharge (p = 0.04). Interpretation of the correlation coefficient indicated that an average daily dose of 1000 mg of gabapentin was associated with a reduction of 1 MME per day. Individual regression analysis of pregabalin did not demonstrate a significant reduction in MME. However, when multiple regression analysis of acetaminophen, methocarbamol, gabapentin, pregabalin, and duloxetine was performed, both gabapentin and pregabalin demonstrated a significant reduction in the admission-to-discharge MME (p = 0.021 and p = 0.027, respectively), suggesting a potential interplay between gabapentin and pregabalin.

Several limitations of this study should be noted. First, there was variability in prescribing practices for non-opioid analgesics. This study focused on the five most commonly prescribed medications in the cohort: acetaminophen, methocarbamol, gabapentin, pregabalin, and duloxetine. Even within these five medications, acetaminophen, methocarbamol, and gabapentin were more frequently used, whereas pregabalin and duloxetine were less commonly prescribed. Because gabapentin is historically used for amputation-related neuropathic pain, the sample size calculation was based specifically on gabapentin. Consequently, the non-significant findings for pregabalin and duloxetine may reflect insufficient statistical power for these less frequently used medications. This potential underpowering may explain the discrepancy in pregabalin’s statistical significance when analyzed independently versus as part of the multiple regression model. Future studies should specifically evaluate less-utilized non-opioid analgesics and their effect on opioid requirements.

Another limitation is the generalizability of results across all lower extremity amputations. Participants in this study had a variety of amputation etiologies, including traumatic, vascular, and infectious causes, each of which may influence postoperative pain management. Future research could stratify patients based on amputation etiology to determine whether these subgroups respond differently to their pain regimen.

## Conclusions

Postoperative pain control in lower extremity amputees is a clinically important area of research to minimize the side effects of pain medications, particularly opioids. Based on the findings of this single-institution retrospective review, clinicians aiming to reduce opioid dosing in the postoperative period should consider prescribing gabapentin over acetaminophen, pregabalin, methocarbamol, or duloxetine. Further research is warranted on less commonly prescribed non-opioid analgesics, particularly pregabalin. Additionally, subgroup analyses based on the etiology of amputation may help determine whether different patient populations respond differently to specific analgesic regimens.
